# Role of Bifidobacteria on Infant Health

**DOI:** 10.3390/microorganisms9122415

**Published:** 2021-11-23

**Authors:** Silvia Saturio, Alicja M. Nogacka, Guadalupe M. Alvarado-Jasso, Nuria Salazar, Clara G. de los Reyes-Gavilán, Miguel Gueimonde, Silvia Arboleya

**Affiliations:** 1 Department of Microbiology and Biochemistry of Dairy Products, Instituto de Productos Lácteos de Asturias (IPLA-CSIC), 33300 Villaviciosa, Spain; silvia.saturio@ipla.csic.es (S.S.); alicja.nogacka@ipla.csic.es (A.M.N.); gpemonserratjasso@gmail.com (G.M.A.-J.); nuriasg@ipla.csic.es (N.S.); greyes_gavilan@ipla.csic.es (C.G.d.l.R.-G.); 2 Diet, Human Microbiota and Health Group, Institute of Health Research of the Principality of Asturias (ISPA), 33011 Oviedo, Spain

**Keywords:** bifidobacteria, gut microbiota, health, development, early life, infant, probiotics, disease

## Abstract

Bifidobacteria are among the predominant microorganisms during infancy, being a dominant microbial group in the healthy breastfed infant and playing a crucial role in newborns and infant development. Not only the levels of the *Bifidobacterium* genus but also the profile and quantity of the different bifidobacterial species have been demonstrated to be of relevance to infant health. Although no definitive proof is available on the causal association, reduced levels of bifidobacteria are perhaps the most frequently observed alteration of the intestinal microbiota in infant diseases. Moreover, *Bifidobacterium* strains have been extensively studied by their probiotic attributes. This review compiles the available information about bifidobacterial composition and function since the beginning of life, describing different perinatal factors affecting them, and their implications on different health alterations in infancy. In addition, this review gathers exhaustive information about pre-clinical and clinical studies with *Bifidobacterium* strains as probiotics in neonates.

## 1. The Genus *Bifidobacterium*: A Landmark of the Healthy Breastfed Infant

Correct gut microbiota acquisition during the early stages of life constitutes one of the critical processes that will determine the later health of the individual [1,2]. Among the microorganisms present in this early life microbiota, the genus *Bifidobacterium* plays a crucial role, having an important function in newborns and infant development [1]. Bifidobacteria were first described at the beginning of the 20th century by Tissier (1900) (originally named *Bacillus bifidus*) after isolating them from breastfed infant feces [3]. Later, they were included in the family *Lactobacillaceae* (*Lactobacillus bifidum*) until 1924, when *L. bifidum* was reclassified as the new genus *Bifidobacterium* by Orla-Jensen [4]. The genus *Bifidobacterium* belongs to the phylum Actinobacteria and currently comprises 94 recognized (sub)species; most of them are normal inhabitants of the gastro-intestinal tract of humans and animals [5]. They are strictly anaerobic, although some species tolerate moderate oxygen levels [6]. The genus comprises high G+C Gram-positive, non-spore-forming, non-motile, and non-filamentous polymorphic rod-shaped bacteria, which can show a large variety of branchings, with bifurcated or spatulated cellular ends being the most common shapes [7]. During recent years, this genus has been extensively studied due to both its important role within the human intestinal microbiota and the extensive use of certain *Bifidobacterium* strains as probiotic products [5,8,9,10].

In the human gut, bifidobacteria are among the predominant microorganisms during infancy, being the dominant microbial group in healthy breastfed infants [1,11]. At this stage, the microbiota is composed mainly of Actinobacteria, the genus *Bifidobacterium* being the main representative, with relatively high levels of Proteobacteria as well [1]. Later, with the introduction of complementary foods and breastfeeding cessation, they are replaced as a dominant microbial group by other microorganisms [12]. The characteristic microbiota of adults is dominated by the phyla Firmicutes and Bacteroidetes and during this period of life, bifidobacteria remain relatively stable trending to a decrease at senescence [13,14,15]. 

It is generally accepted by the scientific community that the gut microbiota composition, and consequently the dominance of specific species of the genus *Bifidobacterium*, of healthy full-term, vaginally delivered, and exclusively breastfed infants is the standard for healthy infant microbiota development [16]. Traditionally, the higher level of bifidobacteria observed in breastfed infants has been attributed to the presence of bifidogenic oligosaccharides in breast milk [17,18]. However, breast milk also contains commensal bacteria (staphylococci, streptococci, lactic acid bacteria, and bifidobacteria) [19,20,21,22], which may also play a role in the dominance of this microorganism observed in breastfed infants. Moreover, the fact that breast milk promotes correct neonatal microbial and immune system development, prevents several non-communicable diseases, and improves cognitive outcomes [23,24,25,26] has prompted the industry towards the supplementation of infant formulas with these microorganisms. 

The early life dominance of bifidobacteria is of relevance since these microorganisms are considered beneficial and reduced levels of them have been associated with poor health or disease conditions [10,27], including immune and metabolic disorders [28,29,30]. Not just the alteration on the genus *Bifidobacterium* levels, but also on the profile and quantity of the different bifidobacterial species present has been demonstrated to be of relevance to health [10,31,32]. Moreover, the species *Bifidobacterium adolescentis*, *Bifidobacterium animalis*, *Bifidobacterium bifidum, Bifidobacterium breve*, and *Bifidobacterium longum* have the Qualified Presumption of Safety (QPS) status granted by the European Food Safety Authority (EFSA) [33], which has promoted the use of some bifidobacterial species as probiotics in different food products and supplements by the industry.

Some bifidobacterial strains have showed resistance phenotypes to different antibiotics, such as aminoglycosides, metronidazole, mupirocin, streptomycin, polymyxin B, erythromycin, or tetracycline. Some of these resistances are intrinsic due to a lack of cytochrome-mediated drug transport or the presence of an atypical isoleucyl-tRNA synthetase, which represents a low risk of transferability. In other cases, the resistance is because of punctual mutations in specific genes and the risk of transferability remains low. However, the resistance to erythromycin or tetracycline, for example, has been found in transposases or in genes flanked by transposases. Notwithstanding, most of the *Bifidobacterium* strains are susceptible to macrolides, vancomycin, beta-lactams, rifampicin, or chloramphenicol [34].

The species most commonly present in humans include *B. adolescentis*, *Bifidobacterium angulatum*, *B. bifidum*, *B. breve*, *Bifidobacterium dentium*, *Bifidobacterium catenulatum*, *Bifidobacterium pseudocatenulatum*, *B. longum*, and *Bifidobacterium pseudolongum* [35], whereas *B. animalis* subsp. *lactis* is the species more often included in functional foods and food supplements [36]. Interestingly, some species, such as *B. adolescentis*, *B. pseudocatenulatum*, or *B. catenulatum*, are often more abundant in adults [37,38,39]. In babies, *B. longum* subsp. *longum* and *B. longum* subsp. *infantis*, together with *B. breve* and *B. bifidum*, are the most commonly found, although the *Bifidobacterium* species composition will be affected by different perinatal factors [32,40]. *B. longum* subsp. *longum* is the species that predominantly inhabits both the infant and adult intestines [41]. It is also important to underline that bifidobacteria show a strong ecological specialization that restricts these microbes to the animal gastrointestinal system; moreover, species specialization seems to exist, with the species distribution depending on environmental factors, host age, and localization in the intestine [32,35,37,42].

The moment at which the microbiota, in general, and bifidobacteria in particular, colonize the human gut is still controversial. Some authors have suggested that colonization may start in utero [43,44], whereas others suggest that colonization starts at delivery [45,46]. In any case, after a very early initial colonization likely led by facultative anaerobic and aerotolerant microorganisms that last for just few hours, *Bifidobacterium* becomes a dominant genus in the newborn microbiota [12,32]. During recent years, several studies have reported the vertical transmission of bifidobacterial strains from mother to infant [47,48,49], the main bifidobacterial colonization occurring from birth and being influenced by several intrinsic and extrinsic factors [10,50].

It is of relevance to underline that an aberrant bifidobacterial number or composition is perhaps the most frequently observed intestinal microbiota alteration in infant diseases. However, no definitive proof is still available on the causal association between reduced bifidobacterial levels, or altered species composition, and disease. This suggests an important role of bifidobacteria in the intestinal homeostasis, perhaps with a causal role in some diseases or as a marker of disrupted homeostasis in others.

## 2. The Genus *Bifidobacterium* and Neonatal Health

Mounting evidence states that there is a critical window for later health in early life when the establishment and composition of the gut microbiota is pivotal for immunological development and establishment of physiological homeostasis. This established concept, called developmental origin of health and disease (DOHaD), spans a period from conception to 2 years of life, the so-called ‘first 1000 days’, in which any particular set of environmental factors produce fingerprints on the genomes (encompassing microbiome and host genome), programming health and the future risk of illness of an individual for life [51]. However, it is known that particularly during the first three months of life is when aberrancies on the gut microbiota establishment are most significant in impacting the immune system development, and consequently future health [24]. Several studies carried out both in animal models and in human clinical trials have demonstrated the importance of the *Bifidobacterium* genus in the very earliest stages of life and reduced levels of them have been associated with disease, as it was stated above [10,24]. Thus, exposure to suitable factors during the initial stages of life is crucial and it is of great interest to unveiling how gut microbiota, and in particular the bifidobacterial community, is shaped by them in this period. 

### 2.1. Starting Foundation of Health: Bifidobacteria in the Dyad Mother-Infant

Mothers provide the first microbial inoculum for the child and this fact influences the later health. As babies pass through the birth canal, they come in close contact with several members of their mother’s vaginal, skin, and fecal microbiota, with this being considered by the scientific literature as the colonizing inoculum, which will give rise to a healthy infantile intestinal microbiota, if the conditions are optimal [1]. Over the last few years, numerous studies have supported the hypothesis of microbiota’s vertical transmission from mother to children [47,48,49,52,53,54]. Makino et al. explored the relationship between mother and infant intestinal bifidobacteria by using multilocus sequence typing and observed that five bifidobacterial strains were monophyletic between individual mother and infant pairs and were found over time in infant fecal samples [48,55]. Moreover, they described that the former strains were only present on vaginally delivered infants, suggesting that delivery mode may influence the mother-to-infant transmission of strains. Using a more exhaustive technique by sequencing the 16S–23S rRNA gene internal transcribed spacer, other authors observed that mothers and infants shared identical bifidobacterial strains and bifidobacterial communities [49,56]. In particular, two strains belonging to *B. breve* and *B. longum* subsp. *longum* were observed to be transferred from mother to infant and persisted over six months of life. Using a combination of strain-specific pangenome-based metagenomics and genetics-based methods, the same sequence identity on different *Bifidobacterium* strains in the infant–mother dyad was also observed, such as with the species *B*. *bifidum* and *B. longum* [57,58]. The role of breastfeeding in this transmission has also been underlined. Different studies have shown that the same bifidobacterial species present in breast milk were found in the feces of breastfed infants over time [59,60]. Cortés-Macías et al. [61] recently studied pre-gestational body mass index, weight gain during pregnancy, and breastfeeding practices as factors affecting breast milk’s profile variability. Their results showed higher levels of bifidobacteria in average-weight women who exclusively breastfed their infants. In another study, the maternal dietary profile was assessed, observing that higher fiber and carbohydrates intake was related to a higher relative abundance of the genus *Bifidobacterium* in breastmilk [62]. These studies demonstrate that different factors can alter the bifidobacterial transmission through the breastmilk to infants, this being considered as the optimum feeding mode for neonates. 

Breast milk is not only a source of bifidobacteria, but it also favors its growth by providing bioactive compounds like human milk oligosaccharides (HMOs) [63]. *Bifidobacterium* members are perfectly adapted to the colonic area due to a process of coevolution and they are able to use the diet-derived glycans and host-provided carbohydrates (mucin and HMOs) as a carbon source, which play a pivotal “prebiotic” role promoting the growth of beneficial bacteria and the establishment of correct gut microbiota in early life [64]. The species *B. longum* subsp. *longum*, *B. longum* subsp. *infantis*, *B. bifidum*, or *B. breve*, which are generally predominant in the infant gut, can break down HMOs into mono- and disaccharides, providing these microorganisms with a competitive advantage and contributing to a cross-feeding interaction with other gut microbiota members [65]. Moreover, bifidobacteria produce short-chain fatty acids (SCFAs) by fermentation of HMOs, mainly acetate and formate, but also other acids, such as lactate and 1,2-propanediol, with beneficial effects on the host [66,67,68]. In this sense, in a recent study, Henrick et al. demonstrated that a depletion on the HMOs-utilizing bifidobacterial genes from the fecal microbiome was linked to systemic and intestinal inflammation and immune dysregulation markers at the beginning of life [24]. 

### 2.2. Bifidobacterial Features Contributing to Beneficial Action in the Early Gut

*Bifidobacterium* members carry out their beneficial contribution to the infant by interacting with the gut through different cellular structures, such as surface-associated exopolysaccharides, pilis, or serpins, that allow the adhesion of bifidobacteria to enterocytes and trigger proinflammatory responses that take part in the development of the immature immune system [1,69,70]. *In vitro* assays demonstrated how *Bifidobacterium* strains protect the host by preventing the disruption of the intestinal epithelial barrier and increasing the expression of integrant proteins [71] and by competition for nutritional resources. He et al. [72] observed differences in the adhesion capacity between different species of bifidobacteria, in the context of studying the gut microbiota of infants with allergies. The bifidobacterial community of these infants was dominated by *B. adolescentis*, unlike in healthy children, in which *B. bifidum* was predominant. The results of this study showed that adhesion to intestinal mucus was significantly higher in the group of healthy individuals, favoring the colonization of the gut by bifidobacteria members that consume resources and nutrients, thus limiting pathogen establishment [69]. In addition, some species produce bacteriocins with inhibitory activity against pathogens [73] and thanks to the action of acetic acid produced, it was observed that bifidobacteria are able to protect against infection caused by the pathogen *Escherichia coli* O157: H7, by preventing the toxin from spreading through the bloodstream [74]. Other beneficial effects on infant health include folate production in the intestines or increased immune responses to vaccinations [75,76]. 

### 2.3. Perinatal Factors Affecting Bifidobacteria and Health

**Figure 1:**
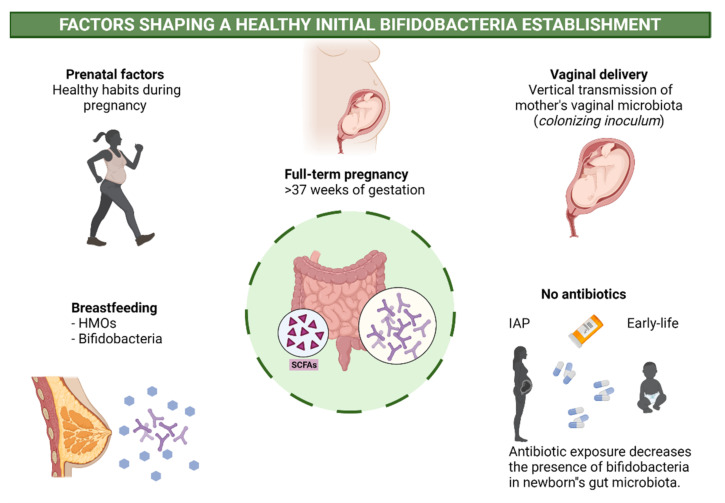
Factors shaping healthy initial bifidobacteria establishment.

Several early life situations may impact early microbiota, and consequently bifidobacteria establishment, influencing later health (Figure 1).

Premature babies (born before 37 weeks of gestational age and/or with very low birth weight–VLBW–< 1500 g) are more likely to have chronic health issues, such as infections (necrotizing enterocolitis–NEC–, sepsis) or asthma, impairment of growth, neurodevelopment or feeding habits, and even an augmented risk of sudden infant death syndrome. Prematurity is one of the factors affecting correct gut microbiota colonization, since consistent observations demonstrated an increased presence of opportunistic and potentially pathogenic microorganisms and decreased levels or delayed establishment of beneficial bacteria, notably of those microorganisms belonging to the *Bifidobacterium* genus [77,78,79,80,81]. It was observed that both in extremely premature (<32 week of gestation) and premature babies (<37 weeks of gestation), *Bifidobacterium* was one of the genera most affected, with reduced levels and abundances during the first three months of life [77,78]. These sorts of differences have been found even at two years of life [82]. A recent study evidenced that prematurity affects bifidobacteria not only at the genus level but also at the (sub)species level; thus, premature babies showed a higher abundance of some specific species, such as *B. breve*, *B. pseudolongum*, or *B. animalis* subsp. *lactis*, along the first trimester of life than full-term infants [32].

The relationship between a low rate of colonization by species of *Bifidobacterium* and the incidence of pathologies, such as NEC or late-onset sepsis (LOS), during the first weeks of life has been reported in different studies [83,84,85,86]. NEC is characterized by an inflamed and necrotic intestine in later stages of the disease and is one of the most lethal diseases in preterm babies, its incidence being inversely related to gestational age and birth weight. Although the etiology of NEC is probably multifactorial and unknown, it appears that there is an inflammation in which the microbiome is involved, and it is known that breast milk protects against the development of NEC in premature babies [87]. NEC and LOS, defined as sepsis occurring ≥ 72 h postnatally, contribute substantially to neonatal morbidity and mortality at neonatal intensive care units (NICUs) and affects mainly premature infants, with serious sequelae [88]. In the study carried out by Stewart et al. [86], a negative correlation was established between the presence of bifidobacteria in the intestinal microbiota of premature children with LOS, whereas the control group (no LOS or NEC) was positively related to the presence of bifidobacteria. Considering that microorganisms causing LOS may be translocated from the intestine [89], the hypothesis defending the protective role of the genus *Bifidobacterium* against infections, such as those mentioned above in premature infants, is reinforced. Moreover, observational studies have linked the loss of *Bifidobacterium* in infants and enteric inflammation early in life [24]. Additionally, some studies associate the prevalence of NEC in premature infants carrying a mutation in the FUT2 gene (non-secretor genotype), which encodes a glycosyltransferase, with a lower abundance of bifidobacteria [90,91].

On the other hand, premature babies suffer a postnatal growth failure that occurs during a critical developmental period of life and the main medical goal is reaching a weight gain and body composition in these infants similar to the fetal intrauterine growth [92]. A positive association was reported between bifidobacterial levels and weight gain in premature babies [93,94]. In a humanized mice model created by fecal transplantation from undernourished infants, *B. longum* was observed to be one of the most discriminatory species correlated with weight gain [95]. This species also showed a negative association with inflammation and anorexia/cachexia mediators in the same previous undernourished infant population [96] and was one of the main conserved structural members in the infant gut microbiota, a core that was defined and used to distinguish different degrees of undernutrition [97].

Delivery mode is known to be a pivotal driver of the gut microbiota composition and can influence the vertical transmission of microbiota at birth from mothers to infants [1,98,99]. While vaginally delivered infants acquire their microbiota from mothers with a less diverse and relatively stable composition overtime, C-section-born babies are susceptible to colonization by more microbes from the environment [1,98]. Despite the variability of the results on gut microbiota composition depending on the way of delivery, several studies have reported a delay in the colonization by bifidobacteria in children born by cesarean section [98,100,101,102], even at one year of life [103]. Moreover, a recent study also observed differences at the *Bifidobacterium* (sub)species level between full-term babies born by vaginal or C-section mode [32]. 

Antibiotic administration during gestation or at early life is another factor known to be able to modify the infant’s gut microbiota. Neonates from mothers that have received intrapartum antibiotic prophylaxis (IAP) administration are characterized by higher levels of Proteobacteria and Firmicutes and lower levels of Actinobacteria and bifidobacteria [104,105]. It was also observed that IAP also affected bifidobacteria at the species level [106]. Antibiotic administration in full-term infants during the first hours of life led to a relative decrease in *Bifidobacterium* and an increase in fecal Proteobacteria [107]. A recent study that evaluated the long-term impact of antibiotic treatment in the neonatal period and early childhood on child growth also reported that neonatal antibiotic exposure is associated with a significantly decreased abundance and diversity of fecal bifidobacteria until two years of age [108]. Of utmost importance is to take into account that early life antibiotic exposure modifies the “natural” progression of the infant gut and the loss of bifidobacteria may lead several negative consequences for infant health including childhood overweight or obesity [109].

## 3. The Genus *Bifidobacterium* and Child Development

Recent evidence suggests that a failure to enhance microbial establishment during early life can result in an increased risk of childhood inflammatory diseases and contributes to an increased obesity risk and metabolic diseases in later life. During the last decade, we have started to understand that the gut microbiota and brain are connected and bidirectionally communicated, and in this two-way path, the gut microbiota can interfere with the normal function of the brain. The importance of understanding the impact of one of the most important members of the early gut microbiota, the *Bifidobacterium* genus, on future child development is also becoming evident.

### 3.1. Bifidobacterium Genus as a Biomarker of Metabolic Diseases in Infanthood

Obesity, particularly in children and adolescents, has become a significant public health problem that has reached epidemic levels worldwide. Recent models have predicted that 57% of today’s children will be obese by the age of 35 [110]. Maternal obesity impacts on the offspring’s gut microbiota and the risk for obesity and non-alcoholic fatty liver disease (NAFLD). A recent *in vivo* study employing germ-free (GF) mice colonized with stool microbes from 2-week-old infants born from obese or normal-weight mothers demonstrated the causative role of maternal obesity-associated infant dysbiosis in childhood obesity and NAFLD [111]. High maternal pre-gestational body mass index (BMI) is associated with an altered infant microbiome from the first days of life until 2 years of age, bifidobacteria being one of the genera most affected, and can also shape the composition and diversity of the breast milk microbiota [61,112,113]. Recent evidence has shown that pre-gestational normal-weight women with exclusive breastfeeding habits harbored a significantly higher abundance of the *Bifidobacterium* genus in the breast milk that could also have an impact on infant development and health [61]. The mother’s BMI was negatively associated with *Bifidobacterium* levels in milk samples and positively associated with colostrum *Lactobacillus* and *Staphylococcus* levels. Therefore, breastfeeding and bifidobacteria in milk provide advantages for both mothers and infants particularly, by decreasing the risk of obesity and other metabolic-related problems [25,60]. 

Caesarean delivery and IAP are well-known risk factors for the gut dysbiosis of early infancy, including a reduced abundance of *Bifidobacterium* and *Bacteroides* [100,114,115], which is associated with greater risk for overweight and obesity [1,116]. However, only few studies have assessed the impact of infant gut microbiota and their metabolites on the disease development. A recent study performed with full-term infants reported that formic acid, a microbial metabolite that can be produced by the *Bifidobacterium* genus, is linked with infant growth. When this metabolite was present at high amounts in 3-month-old infants, it was associated with a higher abundance of *Bifidobacterium* at the age of one year, and subsequently with lower BMI [117]. In addition, obese children have an increased risk of developing obesity-related diseases, such as metabolic syndrome and diabetes, as well as an increased risk of mortality and adverse health outcomes later in life. Decreased numbers of fecal bifidobacteria have been detected in early infancy in those who later go on to become obese, in comparison with healthy children [118]. A prospective study reported that bifidobacterial levels were significantly lower at 6 and 12 months, and specifically *B. longum* and *B. breve*, in children who at seven years of life displayed overweight, and the administration was able to reduce serum and liver triglyceride levels and to decrease hepatic adiposity [28]. Murri et al. observed a dysbiosis in the gut microbiota of diabetic children with respect to healthy children, with *Bifidobacterium* levels significantly decreased and negatively and significantly correlated with the plasma glucose level [119]. Therefore, the above results suggest that bifidobacterial species intervene and are determinant in the mentioned infant metabolic health risks [120]. These data point out at *Bifidobacterium* as a potential early life biomarker to assess the risk of overweight, obesity, and metabolic diseases associated in infants.

### 3.2. Maturation of the Immune System: Early Bifidobacterial Alterations Leading to Atopy

Atopic diseases are the most common allergic conditions in children [121]. The atopy is characterized by an exaggerated IgE-mediated immune response to allergens that are usually innocuous and clinical manifestations include atopic dermatitis, allergic rhinitis, asthma, and food allergy. The term “atopic march” refers to the common fact that children who have one of these diseases are at high risk for further developing another one during childhood. The atopic march usually follows the order: atopic dermatitis > food allergy > asthma > atopic rhinitis [122]. Atopic diseases have increased in the last decades [121], especially in developed countries. Genetic and environmental factors have a crucial role in this condition; in this regard, the “hygiene hypothesis” [122] assumes that reduced early life exposure to factors contributing to the modulation and maturation of the immune system, as the contact and infection with different microbes could increase the risk of developing atopic diseases [123]. As it is known, the gut microbiota is one of the most important contributors to the development and maintenance of immune system functions. Some studies provide evidence that the cross-talk between the gut microbiota and the human immune system during early life is an important regulator for the later development of atopic and allergic diseases [124,125]. 

The genus *Bifidobacterium* plays a key role in this cross-talk. Several studies have reported that the intestinal microbial colonization pattern differs between non-allergic and allergic infants, with the latter group harboring a lower abundance of *Bifidobacterium* [126,127]. Later on, during childhood, the information is more controversial since some authors evidenced higher levels of the genus *Bifidobacterium* in allergic infants than in healthy children [128,129]. Lower diversity within the genus *Bifidobacterium* has been found in fecal samples of infants suffering cow’s milk protein allergy [130]. The abundancies of some relevant species also differentiate the intestinal bifidobacterial populations of allergic from that of non-allergic children [130,131]. Fieten et al. identified six bacterial species in children with atopic dermatitis, whose profile discriminated individuals also presenting food allergy. Apart from *E. coli*, *Faecalibacterium prausnitzii*, and *Akkermansia muciniphila*, the other three species of this group in this study included *B. breve*, *B. pseudocatenulatum*, and *B. adolescentis* [124]. Toddlers with eczema were observed to harbor significantly lower counts of *Bifidobacterium* [132]. Moreover, Watanabe et al. found significantly lower levels of *Bifidobacterium* in adolescents with atopic dermatitis than in healthy control subjects; these levels were significantly lower in patients with severe skin symptoms than in those with mild skin symptoms [133]. 

Mechanisms linking the maturation of the immune system with early gut *Bifidobacterium* populations are not completely understood yet. However, recent articles shed light on this key aspect that associates the nascent microbiota with human health. Regulatory T cells (Treg) are T cell subsets that promote immunological tolerance and inhibit the development of allergy and autoimmunity. In a prospective study, Ruohtula et al., working with a birth-cohort of Estonian and Finnish children from 3 to 36 months of age, found a relationship between the maturation of the gut microbiota and blood Treg. In their children’s cohorts, the maturation of circulating Treg cells involved a decrease in their frequency together with an increase in highly activated Treg, which was associated with the relative abundance of *B. longum*, followed by the colonization of butyrate-producing bacteria. The delay of this process in the Finnish children cohort comparatively increased the risk of IgE-mediated allergic sensitization with respect to the Estonian children [125]. Much of the suppressive potential of Treg cells depends on the long-term stable expression of FoxP3, a protein that acts as a key regulator transcription factor in activated Treg cells. The regulation of FoxP3 expression in Treg cells is a key step that occurs by complex signaling and epigenetic mechanisms [134], some of which involve bifidobacteria. The ability of some *Bifidobacterium* strains to increase, *in vitro* and *in vivo*, the expression of FoxP3 in Treg cells [135,136,137] mediated by interleukin-10 production [138] has been reported.

### 3.3. Brain Development and Social Behavior: New Target to Study the Significance of Bifidobacterium in Infant Health

Infants exhibit different temperaments early in life and these behavioral traits could be critical in children’s mental developmental pathways and can precede later psychopathology [139]. Some studies indicate that the gut microbiota play essential roles in the neurodevelopment and control of behavior [140]. We are still beginning to understand the role that bifidobacteria could play in neurological development and the temperament and behavior in early infancy. In two recent observational studies carried out in infants under one year of age, the authors reported traits, such as soothability and surgency (positive emotionality), as being associated with a higher relative abundance of the genus *Bifidobacterium* in the gut microbiota [141,142]. In addition, another study identified two bifidobacterial species related to negative emotionality (*B. pseudocatenulatum*) and regulation/orienting (*B. pseudocatenulatum* and *B. catenulatum*) temperamental domains, with both of these traits being marked by an enrichment of the genus *Bifidobacterium* [143]. In older children, with cohorts ranging from 2 to 9 years of age, altered levels of bifidobacteria have been described to be associated with neurodevelopmental disorders, autism, and children with a range of adverse experiences and caregiver stressors [144,145,146]. 

In an autism animal model, the impaired social interactions correlated with a gastrointestinal dysfunction and alterations on the bifidobacterial level, which were also associated with alterations of bile acid and tryptophan metabolism [147]. Luk et al. demonstrated the causal role of *Bifidobacterium* administered in the neonatal period in modifying the behavior of adult gnotobiotic mice. The altered behavioral pattern (abnormal memory, sociability, anxiety-like behavior, and motor performance) of adult mice’s GF has been partly rescued when animals were neonatally colonized with conventional murine microbiota or with a consortium of four *Bifidobacterium* species known to be present at high levels in the commensal microbiota of human infants. The recognition memory was the trait more characteristically improved both in males and females. Conventionalized animals and those receiving the *Bifidobacterium* consortium presented anatomical differences in the central nervous system with respect to GF animals, which point to a homeostatic developmental balance of neural connections in the postnatal time period in re-colonized mice [148]. 

Bifidobacteria inhabiting the infant gut participate in the production of some metabolites that mediate gut–brain communication and are important in neurodevelopment. Some of them are tryptophan (an essential amino acid, present in proteins) and derived metabolites, which are important for intestinal and systemic homeostasis and have a positive impact on neurological functions. The predominant bifidobacterial species in the infant gut are able to produce indol-3-lactic acid (ILA), a tryptophan-derived metabolite, and, particularly, bifidobacteria from the infant gut produce more ILA than bifidobacteria present in the adult’s intestine [149,150]. The induction of neurite outgrowth by ILA in a dose-dependent manner was demonstrated when this compound was added to cultures of PC12 cells, a rat adrenal pheochromocytoma lineage [151]. An anti-inflammatory effect of ILA produced by *B. longum* subsp. *infantis* in the presence of HMOs via activation of the aryl hydrogen receptor and nuclear factor erythroid 2–related factor 2 pathway was also observed [152]. On the other hand, sialic acid (N- Acetylneuraminic acid) is an essential compound for optimal brain development and cognition and takes part of oligosaccharides from human milk. Sialic acid is present in the brain of human beings at a higher concentration than in other mammals and it is also more abundant in the brain of breastfed babies than in those receiving formula [153,154,155]. The comprehension of the metabolism of sialylated oligosaccharides by bifidobacteria in the infant gut could help to improve our knowledge of the role of *Bifidobacterium* in the host–microbiota crosstalk and neurodevelopment processes during early infancy.

## 4. The Genus *Bifidobacterium* as a Probiotic for Infants

Bifidobacteria is widely used as a probiotic for preventive and therapeutic purposes in neonates and infants. Probiotics are defined as live microorganisms, which when administered in adequate amounts confer a health benefit on the host [156]. Historically, the selection of probiotics was based on technological properties; however, nowadays, scientific evidence has demonstrated that the probiotics selection has to be done in a rational way, focused on specific targets and populations [157]. Given all the declarations cited before: high abundance on full-term, vaginally born, and breastfed infants; ability to metabolize HMOs; producers of SCFAs and other beneficial substances; and all the beneficial associations with a healthy status, among others, bifidobacteria are recognized as the ideal microorganisms for the development of probiotics for early life. In the next section, we expose different studies with probiotics both in neonatal animal models and clinical intervention studies with infants. 

### 4.1. Animal Models

As already stated in this review, the natural establishment of the gut microbiota may be disturbed by several perinatal factors leading to a dysbiosis, associated with adverse health-related consequences in the neonatal stage or later in life [50]. The importance of correct timing of neonatal colonization becomes apparent in studies on the restoration of the microbiota in GF animals, with recovered normal function when realized in the period of early life but not in adulthood [158]. This crucial window of opportunity to shape the gut microbiota and consequently future health was carried out with several strains from the *Bifidobacterium* genus in animal models attempting to correct neonatal colonization and mucosal immunity, and to treat various infectious, allergic, neurodevelopmental, and metabolic conditions (Table 1). It should be noted that the vast majority of studies on the functionality and security of probiotics have been conducted in adult animals. Whereas, in the studies mentioned in Table 1, administration techniques specially adapted to the neonatal stage have been used, such as microbial transfer from dams to the offspring, treatment in the pregnancy period, and subsequent cohousing and breastfeeding periods. Alternatively, special cannulas were used, adapting the administrated volume to the animal’s growth. 

Several studies have demonstrated the potential role of *B. breve* (NCIMB8807, M-16V, AH1205) and *B. longum* AH1206 in improving the maturation of the intestinal immune system and promoting bifidobacterial colonization during early infancy [159,160,161,162,163,164]. An extensive transcriptomic regulation involving ≈4000 genes significantly upregulated in the intestinal epithelial cells was observed after 3 days of treatment of two-week-old mice with *B. breve* UCC2003, including key genes linked with epithelial barrier development, and driving transcriptomic alteration [159]. Additionally, an influence on the correct development of the mucosal immunity in early life was observed, by an enhancement of the homing process of naive T lymphocytes to the mesenteric lymph node, and the retention of activated lymphocytes in the intraepithelial compartment [160], reduced expression of inflammation-related genes in the colon [161], and an increased Treg proportion in the large intestine as well as enhanced gut immune and endocrine development in suckling mice [162,163].

The etiopathogenesis of allergic conditions is likely to be multifactorial, although alterations in gut colonization during early life are possible factors promoting abnormal postnatal immune maturation [194]. Studies conducted in an animal model of ovalbumin (OVA) sensitization in the neonatal stage with a previous treatment period with *Bifidobacterium* strains have demonstrated their potential to alleviate the risk of IgE-mediated allergies in adulthood, by exhibiting a minor increase in serum IgE levels induced by intraperitoneal-OVA challenge in the adult period and significantly higher IL-10 levels than in the control group [167]. Additionally, a positive effect of bifidobacteria treatment in models of airway inflammation was demonstrated by a reduction of allergic lung inflammation [168,169]. Regarding food allergy, it was suggested to occur due to defective oral tolerance in the neonatal stage [171], and various preclinical studies of a diet supplemented with prebiotic oligosaccharides and *B. breve* M-16V tested its capacity to satisfactorily prevent allergy development in an early life murine model of orally induced cow-milk allergy [171,172]. 

The neonatal stage is fundamental for proper neuronal development. Experimental studies suggest that the dysregulation of the microbiota–gut–brain axis in early life can impact brain development and behavior, resulting in altered cognitive and anxious phenotypes in adulthood [195]. In this way, several studies found that a mix of bifidobacteria species (*B. longum* subsp. *infantis*, *B. breve*, *B. dentium*, and *B. bifidum*) administrated to GF animals restored normal anxiety-like behavior and improved recognition memory [148,175]. Studying acute stress, the exaggerated hypothalamic–pituitary–adrenal (HPA) system stress response in GF mice was partly reversed by reconstitution of the microbiota with bifidobacteria [177]. In a maternal separation-induced stress model, the administration of a strain of *B. pseudocatenulatum* attenuated some aspects of the excessive HPA axis, particularly corticosterone production at baseline and in response to subsequent acute stress in adulthood [176].

Different models of experimental NEC were also subject to extensive investigation with bifidobacterial strains tested as probiotics. A protective effect of *B. infantis* (BB-02, ATCC15697), *B. adolescentis* (n/a), *B. bifidum* (OLB6378), *B. breve* M-16V, and the combination of *B. bifidum* PM-A0218 and *B. longum* PM-A0101 was reported on the intestine of newborn rats and mice, with a reduced NEC incidence and intestinal damage severity [178,179,180,181,182,183,184,185,186,187]. In rotavirus-associated diarrhea, bifidobacteria-treated animals were more strongly protected and presented an earlier resolution of the disease than non-treated animals [188,189,190]. 

### 4.2. Clinical Trials

The beneficial effects of bifidobacteria and the absence of negative effects following their oral administration have prompted their use in infant nutrition to support the development of a healthy microbiota and prevent diseases.

Some studies have evaluated the safety of probiotic formulas in early infancy [196,197] and different studies have reported the benefits of probiotics in reducing the incidence and severity of gastrointestinal infections or constipation (Table 2). The amelioration of acute diarrhea in the pediatric population is one of the best-studied outcomes of probiotics use in general, and particularly of bifidobacteria. The main and consistent positive effects of bifidobacterial strains tested in clinical studies, both individually or in a cocktail of different probiotics, were a reduction of diarrhea duration and/or severity [198,199,200,201,202,203]. For example, different studies have observed that the administration of *B. lactis* BB-12 daily included in an infant formula or yogurt reduced diarrhea episodes, with respect to the placebo or other formulas not containing bifidobacteria [202,203]. Two trials investigated supplementation with several *B. animalis* subsp. *lactis* strains (doses not reported) in healthy infants with human immunodeficiency virus-infected mothers. While one trial reported significantly enhanced head growth and a trend toward increased weight gain compared to placebo [204], the other trial did not observe differences in these parameters between the *B. lactis* strain tested (CNCM I-3446) and placebo groups [205]. 

Secretory immunoglobulin A (sIgA) is one of the most abundant immunoglobulins in the human body and is predominant in mucosal surfaces. sIgA plays a key role in the gastrointestinal defense mechanism against dietary and microbial antigens [206]. Therefore, several trials have been performed with the objective to enhance faecal sIgA through bifidobacterial administration. In a group of infants fed daily with a formula containing *B. animalis* subsp. *lactis* BB12, fecal sIgA was not affected by the treatment [207]; in contrast, a formula containing the same strain administered at a higher concentration to another group of infants significantly improved total sIgA and polio virus-specific IgA [208]. On the other hand, Taniuchi et al. observed that the administration of formulas supplemented with *B. breve* M-16V to infants with cow’s milk hypersensitivity and atopic dermatitis significantly increased the proportion of bifidobacteria in their fecal microbiota and improved their allergic symptoms [209]. Furthermore, the immunostimulation using probiotics was studied in a randomized double-blind placebo-controlled (RDBPC) trial carried out in infants under 5 years administered with a *B. breve* Yakult (BBG-01) in an oral inactivated cholera vaccine. Whereas this strain was well tolerated by children, a post vaccine immunostimulatory effect was not evident [210]. 

As depicted before, premature birth is associated with high mortality rates and several serious complications later in life as well as aberrances in the gut microbiota composition. This highly vulnerable population could greatly benefit from probiotic administration; however, due to their critical immaturity, including an immunodeficient status, particular attention is paid in clinical trials to possible adverse events derived from the administration of these microbial products. A rational use of bifidobacteria given their safety and beneficial effects on early gut microbiota establishment makes them an ideal probiotic option. Several trials have been performed in premature babies with different bifidobacterial strains (Table 3). A trial showed that a formula containing *B. animalis* subsp. *lactis* BB-12 administered daily to premature babies for 1 month improved their gut microbiota composition [211], as well as the combination of *B. bifidum-Lactobacillus acidophilus* in another cohort of preterm babies [212]. A prospective randomized clinical study with VLBW preterm babies examined the effect of *B. breve* YIT4010 supplementation for four weeks [213]. During the administration, the volume of aspirated gas from the stomach was significantly reduced, indicating reduced severity of NEC. The children colonized by *B. breve* YIT4010 received fewer analgesic doses and showed greater weight gain during the four weeks after administration [213]. Similarly, the administration of the same strain (BB-12) to preterm infants in increasing doses reduced intestinal permeability and increased head growth during the first 30 days of life [214]. By contrast, in another clinical trial, the administration of *B. animalis* subsp. *lactis* BB-12 to VLBW infants did not reduce the incidence of nosocomial infections [215]. A large trial carried out in VLBW infants in five different NICUs compared the effect of the administration of a *B. animalis* subsp. *lactis* strain, a prebiotic, and a mix of both (synbiotic) for a maximum of 8 weeks after birth and observed that the NEC incidence was significantly reduced in the *B. animalis* subsp. *Lactis*-administered group. Furthermore, the administration of this probiotic also showed significantly lower rates of nosocomial sepsis and mortality, shorter time to full enteral feeding, and shorter stays in the NICUs [216]. Patole et al. performed a retrospective cohort study in preterm children over two years, and observed a significant reduction of NEC incidence after the introduction of a daily supplement containing *B. breve* M-16V [217]. This effect could be linked to the anti-inflammatory properties of this *B. breve* strain demonstrated in a trial carried out with a group of preterm newborns that received this microorganism daily in two doses, for which increased serum levels of TGF-β compared to placebo-fed infants were detected [218]. However, another multicenter trial, including 24 hospitals analyzing the effect of a daily administration of either a formula containing *B. breve* BBG-001 or placebo in a large number of preterm infants within the first 48 h of birth, did not find statistically significant effects on NEC incidence, sepsis, or mortality rate among groups [219]. Additionally, the early administration of a B. breve strain also had a positive effect on the gut microbiota of low-birth-weight (LBW) babies [220], prevented NEC and infection in extremely-low-birth-weight and VLBW babies [221,222], and reduced the production of butyric acid [223]. In a retrospective analysis of data on small gestational age versus appropriate for gestational age infants for three years, the outcomes between both preterm infants after *B. breve* M-16V administration showed a trend toward a reduction of NEC and LOS in both groups [224]. Additionally, the bacterial cocktail of *B. bifidum-L. acidophilus* and *B. breve-Lactobacillus casei* has been successful in the reduction of NEC incidence [225,226]. Meanwhile, the administration of *B. breve* M-16V promotes bifidobacteria colonization in fecal samples without adverse effects [227,228]. A strain of *B. bifidum* in VLBW infants accelerated the establishment of enteral feeding without increasing morbidity, with a significant decrease in LOS [229], and a pilot study’s administration of *B. infantis* to term babies scheduled for heart surgery within the first few weeks of life did not significantly alter the fecal microbiota and changes in plasma cytokines were found to be inconsistent [230].

In short, most studies carried out until present have shown positive effects of bifidobacteria supplementation in premature infants on the severity and incidence of NEC and infections. Therefore, the treatment of preterm and VLBW infants with bifidobacteria is an alternative therapy that could help on the prevention or treatment of NEC. Despite this, the selection of the appropriate strain and more clinical trials are needed to identify the most effective bifidobacterial probiotics and their potential mechanism of action.

## 5. Conclusions

This review has outlined the pivotal role of the bifidobacteria on the neonatal health. The early gut microbiota is very unstable and is highly susceptible to being affected by external factors, this being especially relevant for the genus *Bifidobacterium* members. Reduced levels of bifidobacteria have been observed in different perinatal circumstances, such as in premature or C-section-delivered babies or after antibiotic treatment, but also in individuals later developing different metabolic, immune, and neurodevelopmental diseases. Nowadays, we are starting to understand that these aberrances not only exist at genus level but also at specific bifidobacterial species levels. The cited considerations, together with the large trajectory and safe use as probiotics, make them ideal candidates to be used in a preventive or therapeutic way in early life when the correct gut microbiota establishment is jeopardized. Several *Bifidobacterium* strains have been studied in pre-clinical models with encouraging results at enteric and systemic levels. In recent years, a substantial number of bifidobacterial strains have already been tested in clinical trials with promising results, especially in premature babies and in diarrheal processes. The evidence discussed in this review suggests a putative causal role of bifidobacteria in some health alterations or diseases and opens the debate to use them as a marker of disrupted homeostasis in early life. In the coming years, novel scientific discoveries will become true and allow a ful understanding of the role of the bifidobacteria function on early human health with important translational deliverables to medical environments and functional food industries.

## Figures and Tables

**Table 1:** Examples of animal studies evidencing the impact of different *Bifidobacterium* strains in early life.

Probiotic Strain	Dose	Target	Animal Model (n)/Start at Postnatal Age/Treatment	Clinical Outcome Results	Refs.
*B. breve* M16V	1 × 10^9^ CFU/mL drinking water	Caesarean section	NIH Swiss mice (n = 6–14)/1 d through nursing dams/daily to 21 d	Restored early life deficit in the *Bifidobacterium* spp. abundance. Restored neonatal recognition abilities and maternal attachment deficits	[102]
*B. breve* UCC2003	2 × 10^6^ CFU	Intestinal barrier	C57BL/6J mice (n = 10)/2 w by oral gavage/3 consecutive days	Changes in neonatal intestinal epithelial cells transcriptome genes/pathways involved in epithelial barrier function	[159]
*B. breve* M-16V	≈4 × 10^7^ CFU	Mucosal immunity	Lewis rats (n = 8)/6 d by oral gavage/daily to 18 d	Improved development of mucosal immunity in early life. Enhanced intestinal IgA synthesis	[160]
*B. breve* M-16V	5 × 10^8^ CFU	Mucosal immunity	F344/Du rats (n = 9–14)/1 d or 21 d by oral gavage/daily 2 w	Reduced expression of inflammatory molecules during the new-born period. Promoted tolerance by unregulated expression of CD3 during the weaning period	[161]
*B. breve* M-16V	5 × 10^7^ CFU + 20 mg oligosaccharides	Mucosal immunity	C57BL6 mice (n = 7–14)/6 d or 14 d by oral gavage/daily 1 w	Differentially expressed genes related to metabolism and immune responses. Enhanced gut immune and endocrine development in suckling mice	[162,163]
*B. longum* AH1206, *B. breve* AH1205	2 × 10^9^ CFU	Mucosal immunity	BALB/c mice (n = 8–10)/1 d by oral gavage/daily 6 w BALB/c and Swiss Webster GF mice (n = 8–10)/6 w by oral gavage/daily 4 w	*B. longum* augmented T reg cell in neonatal and adult mice *B. breve* enhanced the Peyer’s Patch and the splenic T reg cells when administered from birth	[164]
*B. infantis* n/a	n/a	Oral tolerance	BALB/c mice (n = 5)/Neonatal or adolescent by oral gavage/n/a Oral challenge with OVA	Bifidobacteria administered in neonates, but not at a later age, restored the susceptibility of Th2 responses to oral tolerance induction	[165]
*B. infantis* n/a	n/a	Oral tolerance	BALB/c mice (n = 5)/Dams by oral gavage/n/a	Restored oral tolerance at similar levels of SPF mice	[166]
*B. bifidum* TMC3115	1 × 10^9^ CFUfreeze-dried	Allergy	BALB/c mice (n = 54)/1 d by oral gavage/3 w Intraperitoneal OVA challenge	Minor increases in serum IgE levels induced by OVA-challenge in adult stage and significantly higher TNF-α and IL-10 levels	[167]
*B. lactis* BB-12	1 × 10^9^ CFU	Allergy	BALB/c mice (n = 6–9)/1 d by oral gavage/every second day to 8 w of life Aerosolized OVA challenge	Suppression of all aspects of the asthmatic phenotype: airway reactivity, antigen-specific immunoglobulin E production and pulmonary eosinophilia	[168]
*B. breve* M16V	Diet supplemented with 1 × 10^6^ CFU	Allergy	BALB/c mice (n = 6–21)/Pregnant and nursing dams by diet/2 w Exposition to air pollutant and aerosolized	Maternal supplementation with bifidobacteria prevents their offspring from allergic airway inflammation accelerated by the prenatal exposure to an air pollutant aerosol	[169]
*B. longum* subsp. *longum* CCM7952	2 × 10^8^ CFU	Allergy	BALB/c mice (n = 8–12)/mono-associated GF dams by oral gavage/1 dose Subcutaneously sensitization with pollen allergen	Neonatal mother-to-offspring mono-colonization with bifidobacteria significantly reduced the development of allergen-specific immune responses	[170]
*B. breve* M16V	Diet with FOS and 2 × 10^9^ CFU/g	Allergy	C3H/HeOuJ mice (n = 6–8)/3 w by diet/9 d Intradermal whey-challenge	Partial non-responsiveness to whey protein in mice orally exposed to β-lactoglobulin-derived peptides	[171]
*B. breve* M16V	Diet with FOS/GOS and 2 × 10^9^ CFU/g	Allergy	C3H/HeOuJ mice (n = 6)/3 w by diet/7 w Intradermal whey-challenge	Reduction of the allergic effector response in a murine model of IgE-mediated hypersensitivity	[172]
*B. pseudolongum*	2 × 10^7^ cells	Allergy	BALB/c mice (n = 6)/5 w by oral gavage/2 w DNFB-induced contact hypersensitivity model	Reduction in the initial phase of the disease	[173]
*B. bifidum* TMC3115	1 × 10^9^ CFU + antibiotics	Allergy	BALB/c mice (n = 18)/1 d by oral gavage/21 d	Significantly mitigated altered composition of the intestinal microbiota, serum total IgE levels, and the morphology and function of the intestinal epithelium	[174]
*B. dentium* ATCC27678, *B. infantis* ATCC15697, *B. breve* ATCC15698, *B. bifidum* ATCC29521	1.1 × 10^9^ CFU	Neurodevelopment	GF Swiss Webster mice (n = 17)/1 d by oral gavage/every other day to 21 d, when weekly 21 d–6 w	Infant-type *Bifidobacterium* species mimics colonization with a complex microbiota. Restored aspects of normal anxiety-like behavior in a strongly sex-dependent manner. Improved recognition memory	[148,175]
*B. pseudocatenulatum* CECT7765	1 × 10^8^ CFU	Chronic stress	C57Bl/6J mice (n = 18)/2 d by oral gavage/3 w Dairy maternal separation	Attenuation of some aspects of the excessive stress response of the HPA axis, particularly corticosterone production at baseline and in response to acute stress in adulthood	[176]
*B. infantis*	1 × 10^9^ CFU	Acute stress	BALB/c mice (n = 18–24)/mono-associated GF dams by oral gavage/1 dose	Reversed the exaggerated HPA stress response by GF mice	[177]
*B. infantis* (Chr Hansen)	1 × 10^9^ CFU/freeze-dried	NEC	SD rats (n = 24)/1 d by oral gavage/3 d	Reduction in the incidence of NEC	[178]
*B. infantis* BB-02	3 × 10^6^ CFU	NEC	C57BL/6 mice (n = 4–27)/1 d by oral gavage/3 d	Attenuation of the increase in intestinal permeability and decrease of the incidence of NEC	[179]
*Bifidobacterium* sp.	1× 10^10^ CFU/mL in microcapsules	NEC	SPF SD rats (n = 15)/1 d by oral gavage/3 d	Reduced NEC and intestinal damage severity.	[180]
*B. adolescentis*	1 × 10^8^ CFU	NEC	SD rats (n = 15)/1 d by oral gavage/3 d	Prevention of NEC and significantly decreased the rate of NEC-like intestinal injury.	[181]
*B. bifidum* OLB6378	5 × 10^6^ CFU	NEC	SD rats (n = 30)/1 d by oral gavage/4 d	Decreased the incidence of NEC and normalized the expression and localization of tight junction and adherents junction proteins in the ileum	[182]
*B. breve* M-16V	6 × 10^7^ CFU	NEC	SD rats (n = 17)/1 d by oral gavage/4 d	Suppressed the increased expression of molecules related to inflammation and barrier function that resulted from NEC induction	[183]
*B. longum* subsp. *infantis* ATCC15697	5 × 10^6^ CFU	NEC	SD rats (n = 19)/1 d by oral gavage/4 d	Significantly reduced associated inflammation and incidence of NEC	[184]
*B. bifidum* OLB6378	5 × 10^6^ CFU	NEC	SD rats (n = 30)/1 d by oral gavage/4 d	Attenuation of induction of antimicrobial peptides and NEC incidence	[185]
*B. bifidum* PM-A0218 and *B. longum* PM-A0101	1 × 10^8^ CFU	NEC	SD rats (n = 12)/1 d by oral gavage/3 d	Lower mortality	[186]
*Bifidobacterium* sp.	1 × 10^8^ CFU/daily	NEC	SD rats (n = 20)/1 d by oral gavage/3 d	Decreased incidence and reduced the severity of NEC. Inhibition of proinflammatory cytokine secretion and improvement of intestinal barrier integrity.	[187]
*B. breve* YIT4064	0.05% of diet, heat-killed	RV-induced diarrhea	BALB/c mice (n = 39)/dams before and after delivery by diet/9 w /pups 5 d-old challenged with RV	Pups born and nursed by dams fed with bifidobacteria were more strongly protected against RV-induced diarrhea.	[188]
*B. bifidum* ATCC15696 and *B. infantis* ATCC15697	0.75 × 10^8^ CFU/mL and 0.75 × 10^8^ CFU/mL	RV-induced diarrhea	BALB/c mice (n = 35)/1 d by oral gavage/7 w: 1 dose/weekly Pups 5 d-old challenged with RV	Significantly delayed and early resolution of diarrhea	[189]
*B. longum* SPM1206 and *B. longum* SPM1205	Sonicated extract of 2 × 10^8^ CFU	RV-induced diarrhea	BALB/c (n = n/a)/12 d by oral gavage/3 d Previous RV infection	Inhibited rotavirus gene expression and replication with significant increase of IFN-α and IFN-β gene expression	[190]
*B. breve* M16V	2.5 × 10^9^ CFU + starch	Colitis	F344 rats (n = 6–12)/21 d by oral gavage/3 w DSS-colitis	Bifidobacteria modulates normal systemic T-cell immune functions. Under inflammatory conditions ameliorates DSS-induced colitis in weanling rats.	[191]
*B. animalis*	1 × 10^7^ CFU	Pathogen inhibition	C57BL/6 athymic *bg/bg-nu/nu* and euthymic *bg/bg-nu/+* mice (n = 4–27)/Mono-associated dams by oral and anal inoculum/1 dose Infection with *Candida albicans*	Reduced incidence and severity	[192]
*B. infantis* ATCC15697	culture supernatant	Pathogen inhibition	C57BL/6 mice (n = 23–26)/1 d by oral gavage/8 d Infection with *Cronobacter sakazakii*	Protection against *C. sakazakii*-induced intestinal inflammation	[193]

CFU: colony-forming units; d: days; DNFB: 2, 4-dinitrofluorobenzene; DSS: dextran sulfate sodium; f: female; FOS: fructooligosaccharides; GF: germ-free; GOS: galactooligosaccharides; HFD: high-fat diet; HPA: hypothalamic–pituitary–adrenal; m: male; n: sample size; n/a: not available; NEC: necrotizing enterocolitis; OVA: ovalbumin; RV: rotavirus; SD: Sprague–Dawley; SPF: specific-pathogen-free; w: weeks.

**Table 2:** Clinical studies and beneficial effects of *Bifidobacterium* strains on infants.

Probiotic Strain	Dose	Aim	Study Design/Study Population	Clinical Outcome Results	Refs.
*B. longum* subsp. *infantis* CECT7210 (*B. infantis* IM1)	1 × 10^7^ CFU/g formula	To reduce diarrhea incidence in healthy term infants	RDBC (12 w)/n = 190 (age < 3 m)	Reduction diarrhea episodes, well tolerance and lower constipation prevalence	[201]
*B. animalis* subsp. *lactis* BB-12	1.5 × 10^8^ CFU/L milk formula supplemented	To prevent acute diarrhea	RCT (52 w)/n = 90 (age < 8 m)	Reduced risk of diarrhea by a factor of 1.9 (range, 1.33–2.6)	[202]
*B. animalis* subsp. *lactis*	3.6 × 10^7^ CFU/g formula	To determined growth and stool characteristics	Double-blind study/ (up to 4-m age) n = 88 (age 6–10 d)	Protection against diarrheal illness	[203]
*B. animalis* subsp. *lactis*	1 × 10^7^–10^8^ CFU/g formula	To study the growth of HIV-exposed uninfected infants	RDBPC (4 m)/n = 131 (age 37–42 w)	Well growth, increased head growth and a trend towards increased weight gain	[204]
*B. lactis* CNCM I-3446	67 kcal/100 mL formula *Ad libitum*	To study the growth of HIV-exposed uninfected infants	RDBPC (6 m)/n = 132 (age 37–42 w)	Growth and metabolism in HIV-negative infants fed were not affected	[205]
*B. animalis* subsp. *lactis* BB-12	6 × 10^9^ CFU/100 mL formula	To study the effects on the sIgA levels	RDBC (32 w)/n = 57 (age 0–32 w)	Trend towards higher fecal sIgA levels—statistically significant at the age of 16 weeks	[207]
*B. animalis* subsp. *lactis* BB-12	1 × 10^6^ CFU/g formula	To study the effects on intestinal immunity and inflammation	Prospective RDBC (6 w)/n = 172 (age 6 w)	Increment of fecal sIgA	[208]
*B. breve* M-16V	5 × 10^9^ CFU/g cow’s milk	To elucidate the effect on the intestinal microbiota of infants with cow’s milk hypersensitivity and atopic dermatitis	RCT (12 w)/n = 17 (age 2–5 y)	Improvement in the allergic symptoms and increment of bifidobacteria in feces	[209]
*B. breve* strain Yakult (BBG-01)	4 × 10^9^ CFU/g	To enhances the immunogenicity of oral cholera vaccine	RDBPC (4 w)/n = 128 (age 2–5 y)	Well tolerance. Post vaccinal immunostimulatory effect was not evident	[210]

CFU: colony-forming units; d: days; FCIC: functional chronic constipation; HIV: human immunodeficiency virus; m: months; RDBC: randomized doble-blind controlled clinical trial; RDBPC: randomized double-blind placebo-control trial; RCT: randomized controlled trial; w: weeks; y: years.

**Table 3:** Clinical studies and beneficial effects of *Bifidobacterium* strains on preterm infants.

Probiotic Strain	Dose	Aim	Study Design/STUDY Population	Clinical Outcome Results	Refs.
*B. lactis* BB-12	2.9 × 10^9^ CFU/g formula	To reduce the potentially harmful bacteria	RDBPC (1 m)/n = 69 (GA < 37 w)	Bifidobacteria increment. Enterobacteria and clostridia reduction.	[211]
*B. bifidum* and *L. acidophilus*	2.0 × 10^9^ CFU/twice day	To explore the gut microbiota composition and fecal metabolome	Observational study (99 d)/n = 101 (GA < 34 w)	Predominance of *Bifidobacterium* and a lower abundance of pathobionts. Higher fecal acetate and lactate	[212]
*B. breve* YIT4010	5.0 × 10^9^ CFU/day	To investigate the colonization with *B. breve*	RCT (4 m)/n = 91 (GA 25–28 w)	*B. breve* colonization. Fewer abnormal abdominal signs. Better weight gain.	[213]
*B. lactis* BB-12	2.0 × 10^7^ CFU/g of dry milk	To determine the effect on intestinal permeability, somatic growth, tolerance, rates of sepsis and NEC	Prospective randomized case–control study (30 d)/n = 41 (GA 27–37 w)	Reduced intestinal permeability and increased head growth	[214]
*B. lactis* BB-12	1.2 × 10^10^ CFU/kg/day	To reduce the incidence of nosocomial infections	RCT (6 w)/n = 183 (GA 23–26 and 27–29 w)	The incidence was not reduced, and no adverse effect was observed	[215]
*B. lactis*	5.0 × 10^9^ CFU/day	To prevent NEC	Prospective RCT (8 w)/n = 400 (GA 28.8 ± 1.9 w)	Significant reduction in the incidence of NEC	[216]
*B. breve* M-16V	3.0 × 10^9^ CFU/day	To reduce the incidence of NEC	Retrospective cohort study (over the course of 2 y)/n = 1755 (GA < 34 w)	Decrease incidence of NEC ≥ Stage II or all-cause mortality	[217]
*B. breve* M-16V	1.0 × 10^9^ cells/0.5 mL/twice a day	To examine the effect on the immunologic system in relation to TGF-β	RCT (59 ± 29.3 d)/n = 19 (GA 31.3 ± 3.16 w)	Up-regulation of TGF-β1 signaling and attenuation of inflammatory and allergic reactions	[218]
*B. breve* BBG-001	8.2–9.2 × 10^10^ CFU	To reduce NEC, LOS, and death in preterm infants	Multicenter blinded randomized controlled phase 3 study (36 w)/n = 1315 (GA 23–30 w)	There was no evidence of benefit for this intervention in this population	[219]
*B. breve*	1.6 × 10^8^ CFU/twice a day	To evaluate positive effect on gut microbiota	RCT (7 w)/n = 30 (GA 27.8–37.6 w)	Promotion on *Bifidobacterium* colonization and normal gut microbiota development	[220]
*B. breve* M-16V	1.10 × 10^9^ CFU/day	To prevent NEC and infection	Control study (4 y)/n = 338 (GA 27–36 w)	NEC and infection were prevented	[221]
*B. breve* M-16V	1 × 10^9^ CFU/g	To prevent infection and sepsis	RCT (91.8 ± 54.1 d)/n = 108 (GA 28.1 ± 2.8 w)	Development of infections and sepsis were significantly lower	[222]
*B. breve* M-16V	1.6 × 10^8^ CFU twice daily until discharge	To determine the effects on fecal lactic acid and SCFAs	RCT (4 w)/n = 66 (GA 23–36 w)	Butyric acid was reduced	[223]
*B. breve* M-16V	3 × 10^9^ CFU/day	To compare clinical outcomes between preterm SGA vs. AGA infants after probiotic administration	Retrospective cohort study (3 y)/n = 1380 (GA < 34 w)	NEC, LOS, and all-cause mortality rates were similar in preterm SGA vs AGA infants	[224]
*B. bifidum* and *L. acidophilus*	1 × 10^9^ CFU/125 mg/kg twice daily	To prevent NEC	Prospective, blinded, randomized, multicenter controlled(6 w)/n = 580 (GA < 34 w and <1500 g)	Incidence reduction of death or NEC	[225]
*B. breve* and *L. casei*	3.5 × 10^7^ to 3.5 × 10^9^ CFU	To prevent the occurrence of NEC stage ≥ 2 by criteria of Bell	RDBC (30 d)/n = 231 (GA 29.5 ± 2.5 w)	Reduced the occurrence of NEC (Bell’s stage ≥ 2)	[226]
*B. breve* M-16V	5 × 10^8^ CFU/day	To investigate the effects on the intestinal microbiota	RCT (6 w)/n = 46 (GA 29.9 ± 2.3 w)	Promotion of bifidobacteria colonization and the formation of a healthy microbiota	[227]
*B. breve* M-16V	3 × 10^9^ CFU/day	To increase fecal *B. breve* counts without adverse effects	RDBPC (6 w)/n = 159 (GA < 33 w)	Increased the proportion of neonates with detectable *B. breve*	[228]
*B. bifidum* OLB6378	2.5 × 10^9^ viable cells/500 mg/twice a day	To evaluate the benefit on enteral feeding	RCT study (21 d)/n = 585 (GA 28.6 ± 2.9 w)	Acceleration of the establishment of enteral feeding after birth	[229]
*B. longum* subsp. *infantis* ATCC 15697	4.2 × 10^9^ CFU/two times daily	To investigate the impact on the fecal microbiota and plasma cytokines in neonates with congenital heart disease	RCT (for 8 w)/n = 16 (GA 38.4 ± 1.2 w)	There was no significant alteration in the gut microbiota and plasma interleukin (IL)10, interferon (IFN) ɣ and IL1β levels were higher	[230]

AGA: appropriate-for-gestational age; d: days; GA: gestational age; LOS: late onset sepsis; m: months; NEC: necrotizing enterocolitis; RCT: randomized controlled trial; RDBPC: randomized double blind placebo control trial; SCFAs: short chain fatty acids; SGA: small for a gestational age; w, weeks; y: years.

## Data Availability

Not applicable.
